# Supplementary material for: Dynamic transcriptomic profiles of zebrafish gills in response to zinc supplementation
Source: BMC Genomics. 2010 Oct 11;11:553. doi: 10.1186/1471-2164-11-553 (PMC3091702; doi:10.1186/1471-2164-11-553)
Supplement: Additional file 2 — Interactive Direct Interaction Network representing the molecular interactions between zinc, copper, iron, calcium and proteins encoded by transcripts changed by zinc supplementation. Mini web-site containing index.html and hyperlinked pages in subdirectory describing a Direct Interaction Network automatically generated based on curated interactions contained within the proprietary PathwayArchitect database. Ovals represent proteins and the circles symbolize metal ions. Objects are coloured by their abundance in zebrafish at the time-point they were significantly different from the control is a scale from -4 fold (dark green) to +4 fold (dark red). Where significant differences were found at more than one time-point, the colour overlay shows expression at the first instance. Dark blue squares denote 'binding', and light blue squares 'expression'; green squares stand for 'regulation', green diamonds for 'metabolism', and green circles for 'promoter binding'. Arrow heads indicate directionality of the interaction where annotated. All nodes and edges can be further interrogated by selecting the relative area of the image. [file 1471-2164-11-553-S2.zip › PathwayArchitect Zn xs DIN/456541.html]

# TRANSPORT:

|  |  |
| --- | --- |
| Type | TRANSPORT |
| Effect | None |


---

|  |  |
| --- | --- |
| Score | 0 |


---

|  |  |
| --- | --- |
| Reference Count | 10 |


---

|  |  |
| --- | --- |
| Mechanism | Unknown |


---

|  |  |
| --- | --- |
| Reference:0 || Sentence | "The beta-adrenoceptor agonist isoproterenol (ISO) and the hypothalamic neurohormone corticotropin-releasing factor (CRF) concentration-dependently stimulated the release of beta E-IR from superfused AL quarters and NILs, but not from incubated hypothalamic fragments." |
| PMID | 2525039 |
| Year | 1989 |
| Species | Rat |
| Journal | Biochem Biophys Res Commun |
| RefScore | 1 |
| Source | PArchNLP |
  |
|  |  |


---

|  |  |
| --- | --- |
 Reference:1 || Sentence | "A significant increase of immunoreactive beta-endorphin (IR-beta-Ep) secretion in response to 10(-10) M CRH but not to 10(-11) M CRH was observed in pituitaries from the 15th day of gestation, the earliest day that we tested, whereas 10(-11) M CRH stimulated IR-beta-Ep release from the pituitaries of 17.5-day-old fetuses." |
| PMID | 3261876 |
| Year | 1988 |
| Species | Rat |
| Journal | Regul Pept |
| RefScore | 1 |
| Source | PArchNLP |
  ||  |  |


---

|  |  |
| --- | --- |
 Reference:2 || Sentence | "Dose-related IR-beta-Ep secretions induced by 10(-12) M to 10(-10) M CRH were observed in pituitaries of 19.5- and 21.5-day-old fetuses, and 1-, 3- and 9-day-old newborn pups." |
| PMID | 3261876 |
| Year | 1988 |
| Species | Rat |
| Journal | Regul Pept |
| RefScore | 1 |
| Source | PArchNLP |
  ||  |  |


---

|  |  |
| --- | --- |
 Reference:3 || Sentence | "CRH stimulated not only IR-beta-Ep and IR-adrenocorticotropic hormone (ACTH) but also IR-alpha-melanocyte-stimulating hormone (IR-alpha-MSH) secretions from fetal pituitaries." |
| PMID | 3261876 |
| Year | 1988 |
| Species | Rat |
| Journal | Regul Pept |
| RefScore | 0 |
| Source | PArchNLP |
  ||  |  |


---

|  |  |
| --- | --- |
 Reference:4 || Sentence | "Effects of growth hormone-releasing hormone (GRH) and corticotropin-releasing hormone (CRH) on the release of immunoreactive thyrotropin-releasing hormone (ir-TRH) from the rat hypothalamus in vitro were studied." |
| PMID | 3131150 |
| Year | 1987 |
| Species | Rat |
| Journal | Exp Clin Endocrinol |
| RefScore | 1 |
| Source | PArchNLP |
  ||  |  |


---

|  |  |
| --- | --- |
 Reference:5 || Sentence | "These findings suggest that GRH and CRH inhibit ir-TRH release from the rat hypothalamus in vitro." |
| PMID | 3131150 |
| Year | 1987 |
| Species | Rat |
| Journal | Exp Clin Endocrinol |
| RefScore | 0 |
| Source | PArchNLP |
  ||  |  |


---

|  |  |
| --- | --- |
 Reference:6 || Sentence | "DSIP (greater than or equal to 10(-9) M) inhibited both basal and CRF-induced IR-ACTH release from these cells." |
| PMID | 2558325 |
| Year | 1989 |
| Species | Mouse |
| Journal | Neuroendocrinology |
| RefScore | 2 |
| Source | PArchNLP |
  ||  |  |


---

|  |  |
| --- | --- |
 Reference:7 || Sentence | "In addition, the effect of synthetic rat corticotropin-releasing factor (CRF) and arginine vasopressin (AVP) on IR-DSIP secretion was investigated." |
| PMID | 2558325 |
| Year | 1989 |
| Species | Mouse |
| Journal | Neuroendocrinology |
| RefScore | 1 |
| Source | PArchNLP |
  ||  |  |


---

|  |  |
| --- | --- |
 Reference:8 || Sentence | "CRF and AVP at concentrations of 10(-11)-10(-7)M inhibited release of IR-DSIP from mouse anterior pituitary cells by 63%." |
| PMID | 2558325 |
| Year | 1989 |
| Species | Mouse |
| Journal | Neuroendocrinology |
| RefScore | 2 |
| Source | PArchNLP |
  ||  |  |


---

|  |  |
| --- | --- |
 Reference:9 || Sentence | "Corticotropin-Releasing-Hormone (CRH) test in Cushing's disease stimulated the release of both ACTH and IR-hNT, showing a slightly different pattern of secretion." |
| PMID | 2156920 |
| Year | 1990 |
| Species | Human |
| Journal | J Endocrinol Invest |
| RefScore | 1 |
| Source | PArchNLP |
  |


---

|  |  |
| --- | --- |
